# Supplementary figures and images for: Thermal cycling-hyperthermia in combination with polyphenols, epigallocatechin gallate and chlorogenic acid, exerts synergistic anticancer effect against human pancreatic cancer PANC-1 cells
Source: PLoS One. 2019 May 31;14(5):e0217676. doi: 10.1371/journal.pone.0217676 (PMC6544372; doi:10.1371/journal.pone.0217676)

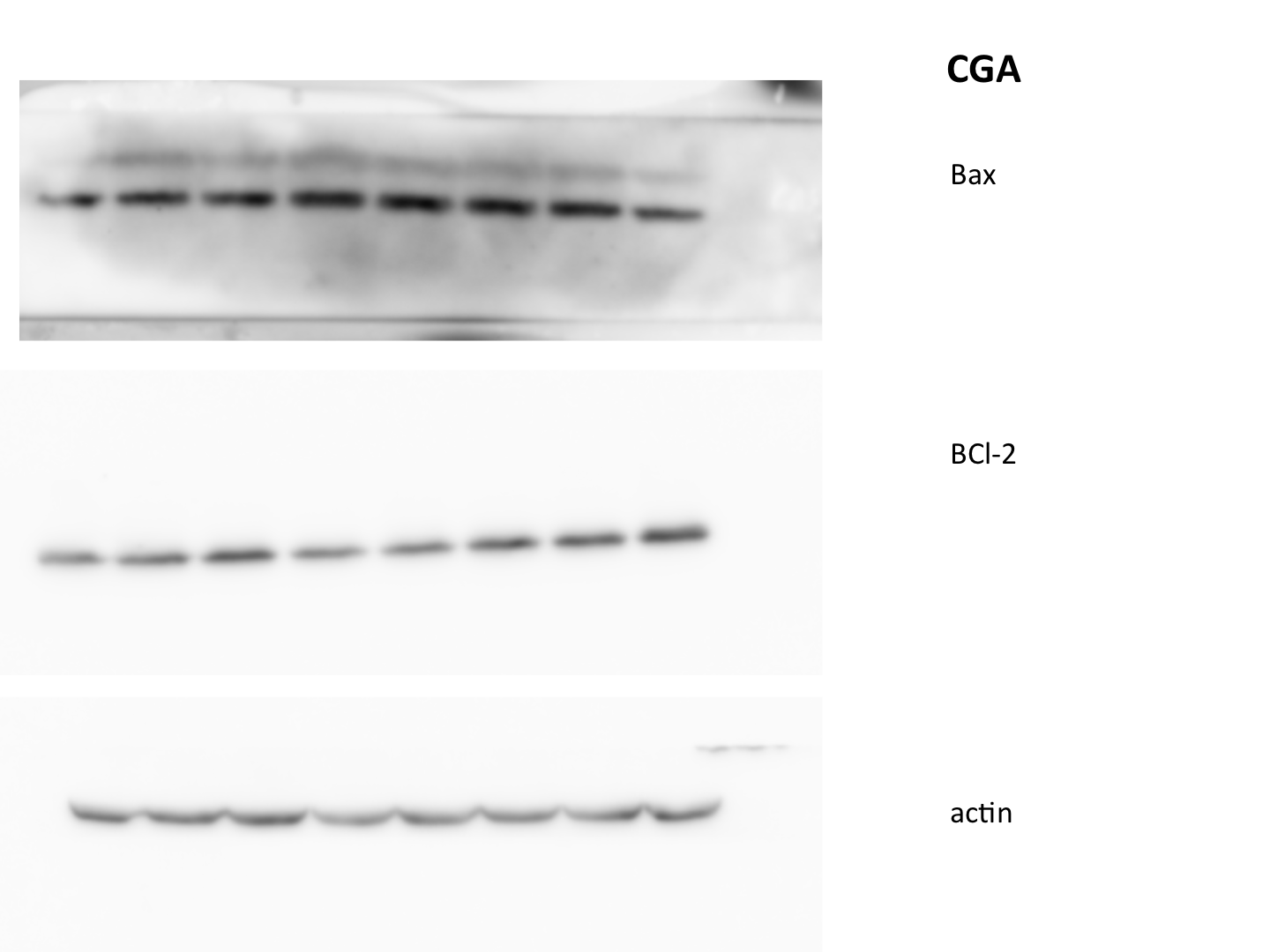

Supplement: S3 Fig — (ZIP) [file pone.0217676.s003.zip › S1_fig/1.tiff]

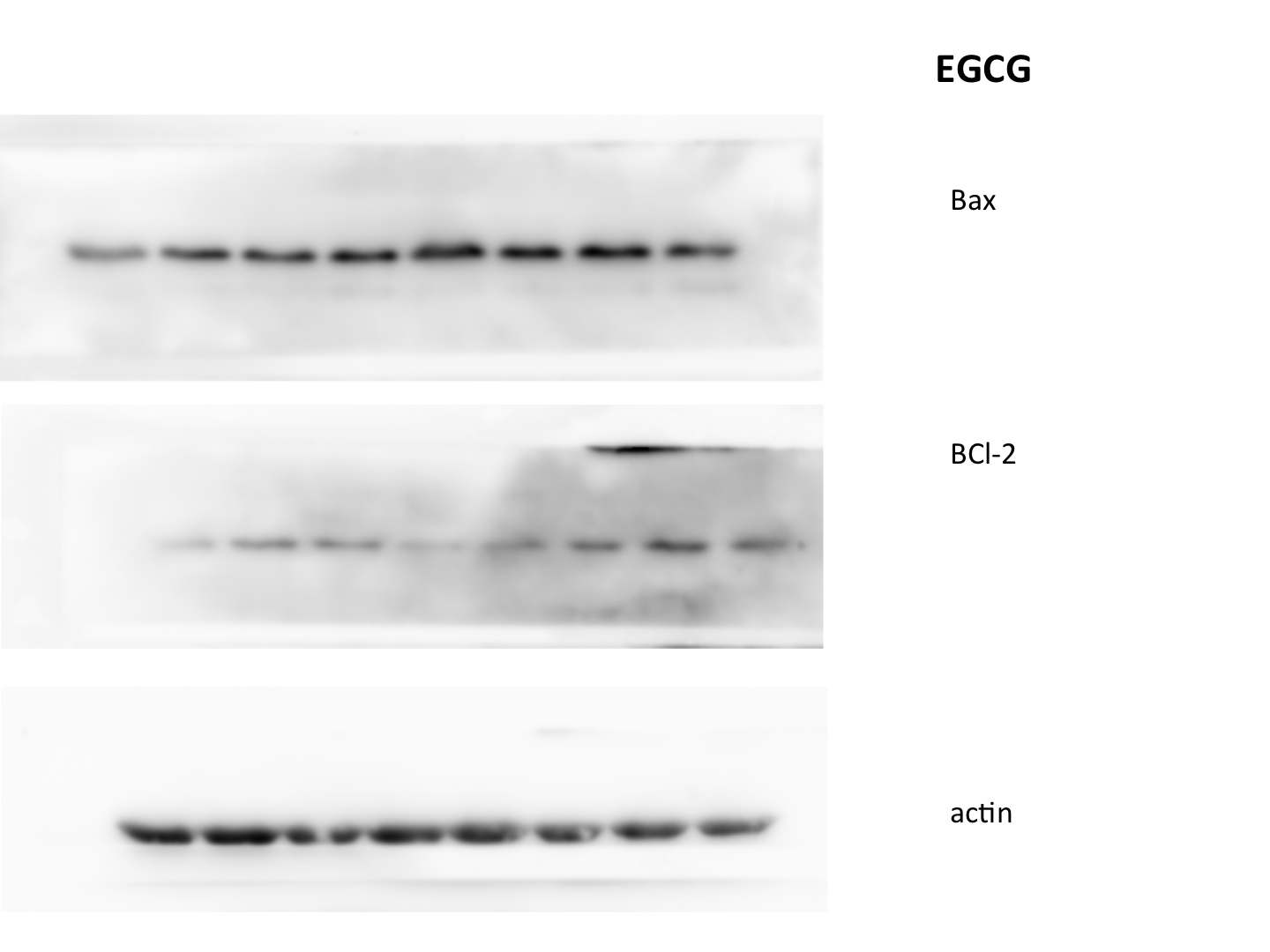

Supplement: S3 Fig — (ZIP) [file pone.0217676.s003.zip › S1_fig/2.tiff]

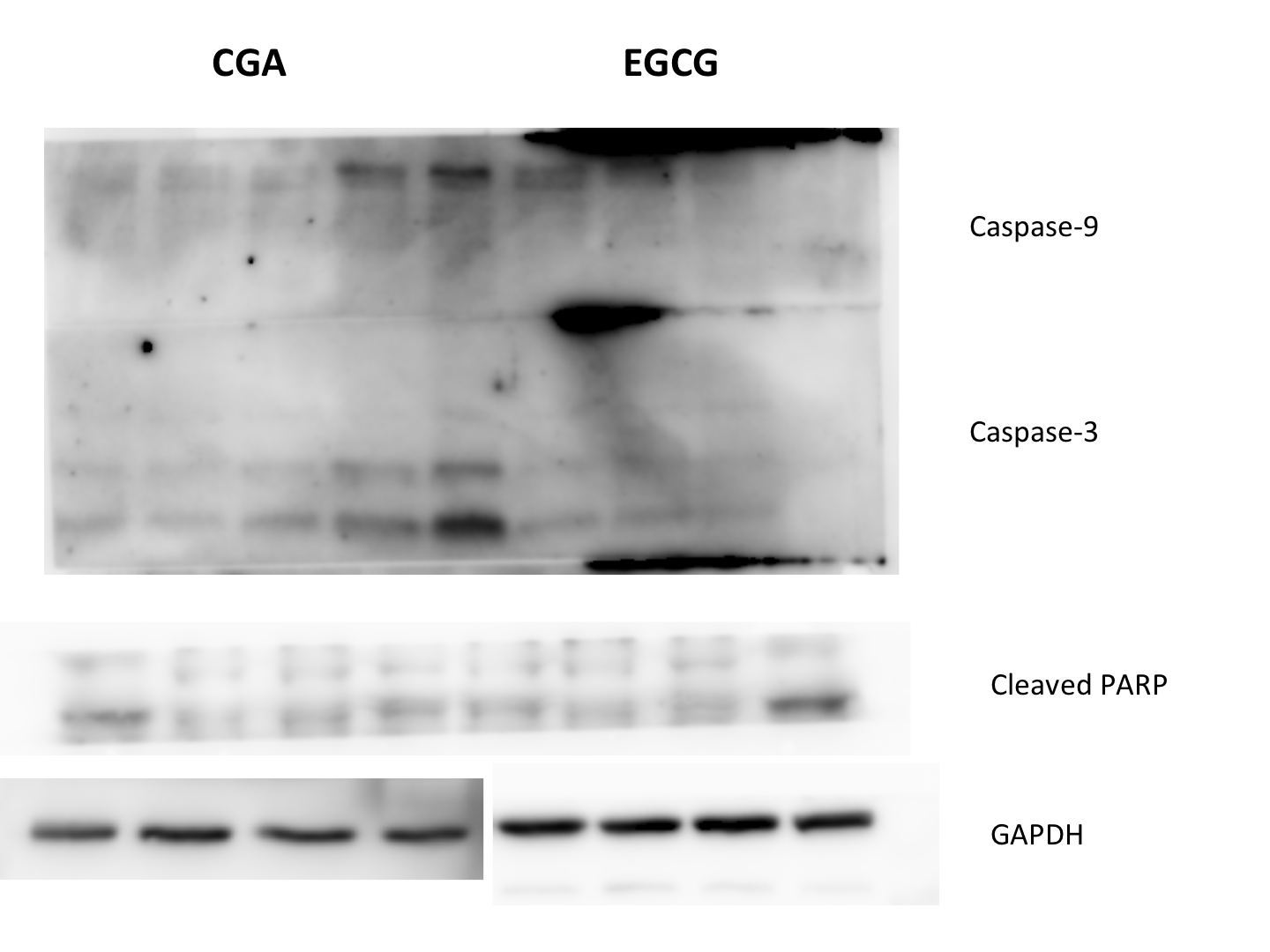

Supplement: S3 Fig — (ZIP) [file pone.0217676.s003.zip › S1_fig/3.tiff]

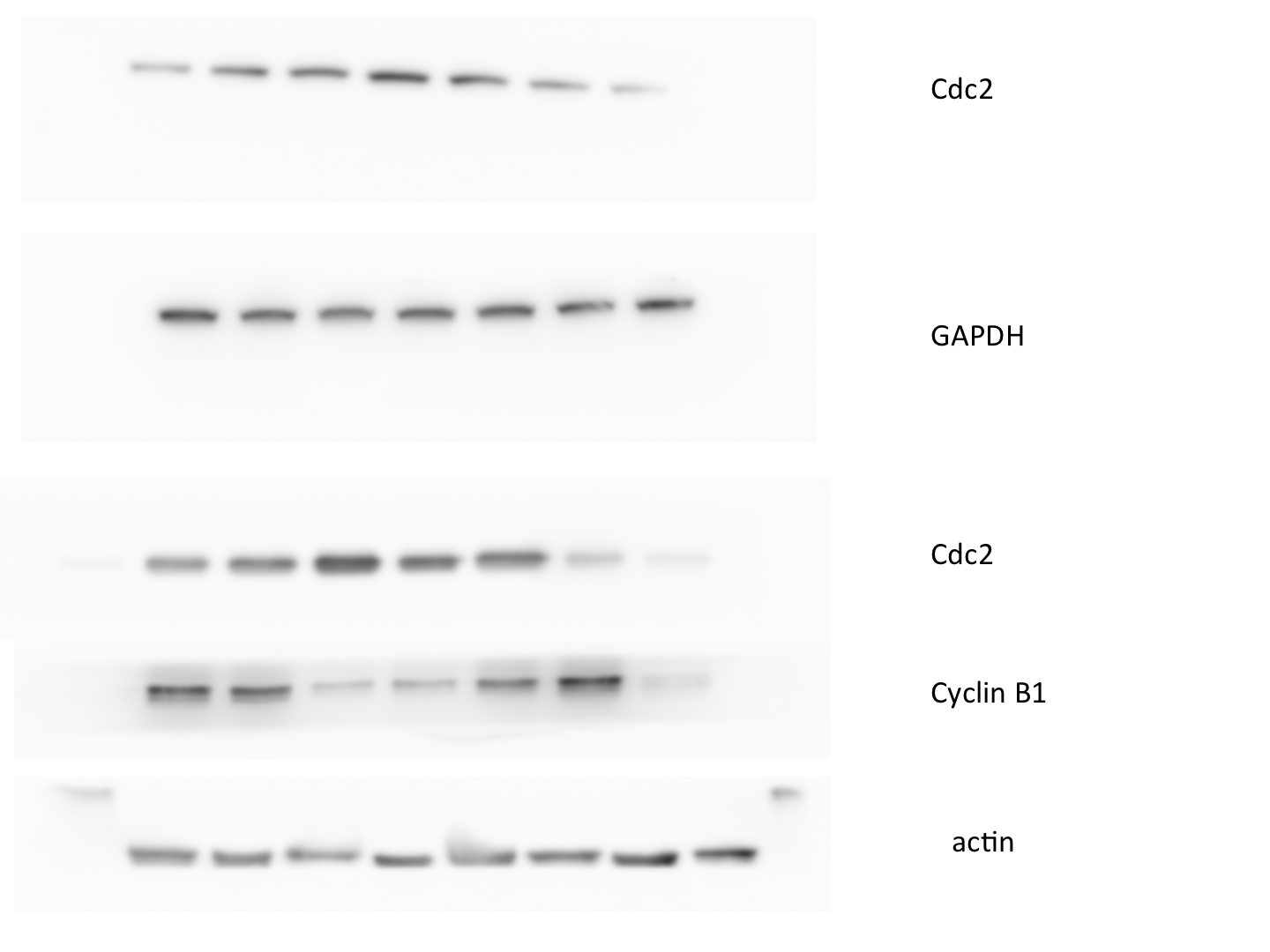

Supplement: S3 Fig — (ZIP) [file pone.0217676.s003.zip › S1_fig/4.tiff]
