# Supplementary material for: Thermal cycling-hyperthermia in combination with polyphenols, epigallocatechin gallate and chlorogenic acid, exerts synergistic anticancer effect against human pancreatic cancer PANC-1 cells
Source: PLoS One. 2019 May 31;14(5):e0217676. doi: 10.1371/journal.pone.0217676 (PMC6544372; doi:10.1371/journal.pone.0217676)
Supplement: S2 File — (PDF) [file pone.0217676.s005.pdf]

Ctrl

	Annexin-/PI-	Annexin-/PI+	Annexin+/PI-	Annexin+/PI+
	93.8	0.9	3.9	1.4
	93.7	0.8	4.3	1.2
	95.4	0.6	3.1	0.9
**Mean**	94.300	0.767	3.767	1.167
**SD**	0.954	0.153	0.611	0.252

DMSO

	Annexin-/PI-	Annexin-/PI+	Annexin+/PI-	Annexin+/PI+
	95.7	1.2	1.5	1.5
	96	1	1.6	1.4
	95.5	2	1.4	1.1
**Mean**	95.733	1.400	1.500	1.333
**SD**	0.252	0.529	0.100	0.208

CGA

	Annexin-/PI-	Annexin-/PI+	Annexin+/PI-	Annexin+/PI+
	87.8	0.9	6.9	4.4
	85.4	0.5	10	4.1
	87.6	0.5	6.9	2.8
**Mean**	86.933	0.633	7.933	3.767
**SD**	1.332	0.231	1.790	0.850

EGCG

	Annexin-/PI-	Annexin-/PI+	Annexin+/PI-	Annexin+/PI+
	89	0.3	8.9	1.7
	91.9	0.4	7	1.5
	89.9	0.5	7.1	2.4
**Mean**	90.267	0.400	7.667	1.867
**SD**	1.484	0.100	1.069	0.473

TC

	Annexin-/PI-	Annexin-/PI+	Annexin+/PI-	Annexin+/PI+
	90	0.6	6.7	2.8
	91.6	1.2	4.3	2.8
	89.8	0.2	7.9	2.2
**Mean**	90.467	0.667	6.300	2.600
**SD**	0.987	0.503	1.833	0.346

DMSO+TC

	Annexin-/PI-	Annexin-/PI+	Annexin+/PI-	Annexin+/PI+	
	95.2	0.9	2.1	1.8	3.9
	92.8	0.8	4.5	1.7	6.2
	93.1	0.6	4.8	1.5	6.3
**Mean**	93.700	0.767	3.800	1.667	5.467
**SD**	1.308	0.153	1.480	0.153	1.358

CGA+TC

	Annexin-/PI-	Annexin-/PI+	Annexin+/PI-	Annexin+/PI+	
	66.6	2.3	19.7	11.4	31.1
	65.3	1.2	12.9	20.6	33.5
	60.4	1.4	23.2	15	38.2
**Mean**	64.100	1.633	18.600	15.667	34.267
**SD**	3.270	0.586	5.237	4.636	3.612

EGCG+TC

	Annexin-/PI-	Annexin-/PI+	Annexin+/PI-	Annexin+/PI+	
	53.8	1.4	24.9	19.8	44.7
	60.4	1.7	24.6	13.3	37.9
	53.1	1.4	23.4	22.1	45.5
**Mean**	55.767	1.500	24.300	18.400	42.700
**SD**	4.028	0.173	0.794	4.564	4.176

C

G0/G1	S	G2/M
54.84	28.67	16.46
59.67	24.56	15.47
58.62	24.68	16.7
57.71	25.97	16.21
2.540334624	2.339038264	0.651996933

DMSO

G0/G1	S	G2/M
56.06	25.52	18.41
51.5	26.18	22.32
51.63	27.34	21.04
53.06333333	26.34666667	20.59
2.596003338	0.921375783	1.993464321

CGA

G0/G1	S	G2/M
59.07	21.89	19.04
55.3	22.56	22.14
53.02	27.09	19.89
55.79666667	23.84666667	20.35666667
3.055426866	2.828715845	1.601821879

EGCG

G0/G1	S	G2/M
50.24	26.64	23.12
55.13	23.91	20.96
54.11	23.27	22.62
53.16	24.60666667	22.23333333
2.579709286	1.789757898	1.130722483

TC

G0/G1	S	G2/M
50.87	19.41	29.72
54.2	16.59	29.2
57.21	24.77	18.02
54.09333333	20.25666667	25.64666667
3.171345666	4.155205571	6.610002521

DMSO+TC

G0/G1	S	G2/M
49.99	27.21	22.8
51.86	24.88	23.26
53.99	23.17	22.84
51.94666667	25.08666667	22.96666667
2.001407838	2.027913542	0.254820198

CGA+TC

G0/G1	S	G2/M
44.35	21.6	34.05
49.74	14.14	36.13
50.85	11.46	37.69
48.31333333	15.73333333	35.95666667
3.476928721	5.254420361	1.826179984

EGCG+TC

G0/G1	S	G2/M
36.8	21.24	41.95
35.98	17.23	46.79
37.21	20.87	41.92
36.66333333	19.78	43.55333333
0.626285345	2.216100178	2.803075692

MMP

C	DMSO	200	20 TC	DMSO+TC	200TC	20TC	
2.1	5.5	3	6.7	13.1	5.5	36.7	68.4
7	3.7	6.2	4.4	3.6	5.5	48.2	75.2
7.5	4.2	4.7	9.6	9.4	7	47.2	83.5
5.533333333	4.466666667	4.633333333	6.9	8.7	6	44.03333333	75.7
2.983845394	0.929157324	1.601041328	2.605762844	4.788527958	0.866025404	6.370504951	7.562407024

ROS

C	DMSO	200	20 TC	DTC	200TC	20TC	
1	1.011862197	1.06366628	1.104932306	1.305825922	1.19640415	2.432041935	3.621156024
0	0.034052381	0.050490275	0.101528241	0.121245507	0.104278388	0.189552541	0.684832326
